# The Gut Microbiome in Sepsis: From Dysbiosis to Personalized Therapy

**DOI:** 10.3390/jcm13206082

**Published:** 2024-10-12

**Authors:** Andrea Piccioni, Fabio Spagnuolo, Marcello Candelli, Antonio Voza, Marcello Covino, Antonio Gasbarrini, Francesco Franceschi

**Affiliations:** 1 Department of Emergency Medicine, Fondazione Policlinico Universitario Agostino Gemelli-IRCCS, 00168 Rome, Italy; andrea.piccioni@policlinicogemelli.it (A.P.); marcello.candelli@policlinicogemelli.it (M.C.); marcello.covino@policlinicogemelli.it (M.C.); francesco.franceschi@policlinicogemelli.it (F.F.); 2 Faculty of Medicine and Surgery, Università Cattolica del Sacro Cuore, 00168 Rome, Italy; antonio.gasbarrini@policlinicogemelli.it; 3 Department of Emergency Medicine, IRCCS-Humanitas Research Hospital, Rozzano, 20089 Milan, Italy; antonio.voza@humanitas.it; 4 Medical and Surgical Science Department, Fondazione Policlinico Universitario A. Gemelli-IRCCS, 00168 Rome, Italy

**Keywords:** intestinal microbiota, sepsis, intestinal barrier, antibiotics, DAO (diamine oxidase), I-FABP (intestinal fatty acid-binding protein), prebiotics, probiotics, symbiotics, postbiotics, FMT (fecal microbiota transplantation), SDD (selective digestive decontamination)

## Abstract

Sepsis is a complex clinical syndrome characterized by an uncontrolled inflammatory response to an infection that may result in septic shock and death. Recent research has revealed a crucial link between sepsis and alterations in the gut microbiota, showing that the microbiome could serve an essential function in its pathogenesis and prognosis. In sepsis, the gut microbiota undergoes significant dysbiosis, transitioning from a beneficial commensal flora to a predominance of pathobionts. This transformation can lead to a dysfunction of the intestinal barrier, compromising the host’s immune response, which contributes to the severity of the disease. The gut microbiota is an intricate system of protozoa, fungi, bacteria, and viruses that are essential for maintaining immunity and metabolic balance. In sepsis, there is a reduction in microbial heterogeneity and a predominance of pathogenic bacteria, such as proteobacteria, which can exacerbate inflammation and negatively influence clinical outcomes. Microbial compounds, such as short-chain fatty acids (SCFAs), perform a crucial task in modulating the inflammatory response and maintaining intestinal barrier function. However, the role of other microbiota components, such as viruses and fungi, in sepsis remains unclear. Innovative therapeutic strategies aim to modulate the gut microbiota to improve the management of sepsis. These include selective digestive decontamination (SDD), probiotics, prebiotics, synbiotics, postbiotics, and fecal microbiota transplantation (FMT), all of which have shown potential, although variable, results. The future of sepsis management could benefit greatly from personalized treatment based on the microbiota. Rapid and easy-to-implement tests to assess microbiome profiles and metabolites associated with sepsis could revolutionize the disease’s diagnosis and management. These approaches could not only improve patient prognosis but also reduce dependence on antibiotic therapies and promote more targeted and sustainable treatment strategies. Nevertheless, there is still limited clarity regarding the ideal composition of the microbiota, which should be further characterized in the near future. Similarly, the benefits of therapeutic approaches should be validated through additional studies.

## 1. Introduction

The human gut microbiota is an intricate system composed of bacterial microorganisms, yeasts, viruses, and parasites. With its various phyla, it reaches a population of nearly 100 trillion microorganisms, predominantly Firmicutes and Bacteroidetes [1]. The human gastrointestinal tract is sterile at the beginning, but it is quickly colonized by the maternal microbiome, with the mode of delivery—vaginal or caesarean—affecting the initial composition of the neonate’s microbiota [2]. The gut microbiota is unique to each individual and influenced by genetic and environmental factors, diet, and antibiotic use [3]. The major bacterial components of the gut microbiota include Firmicutes, Bacteroidetes, Actinobacteria, and Proteobacteria; additionally, Fusobacteria, Cyanobacteria, and Verrucomicrobia are present, totaling seven phyla.

The Firmicutes phylum (60% of the total) is primarily composed of obligate or facultative anaerobic gram-positive bacteria, such as *Enterococcus* spp., *Clostridium* spp., and *Lactobacillus* spp. The Bacteroidetes phylum (30–40%) is mainly composed of gram-negative anaerobic bacteria like *Prevotella* spp. and *Bacteroides* spp. [3] These commensals are crucial as they help ensure host immunity and regulate various metabolic functions such as digestion, nutrient absorption, vitamin synthesis, and energy production. Beneficial commensal microbes can restore proper intestinal barrier function and exert anti-inflammatory effects [4].

**Figure 1:**
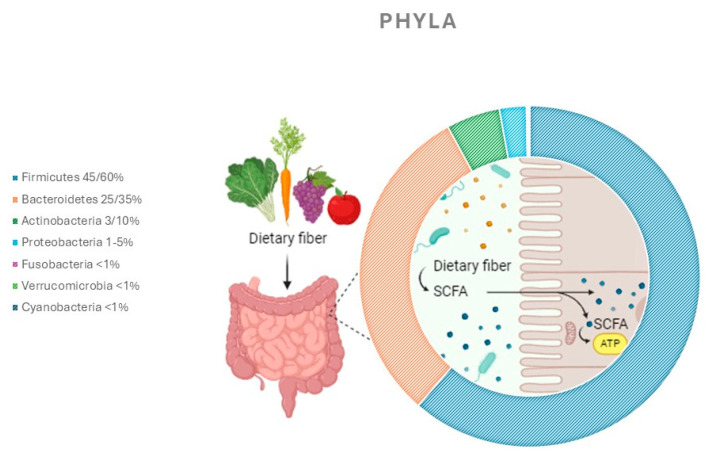
Representation of bacterial phyla in the healthy gut microbiota. Created with BioRender.com.

Dietary carbohydrates are the primary energy source and are fermented by colon bacteria such as *Bifidobacterium* and *Fecalibacterium* to produce SCFAs like acetate and butyrate, which are significant energy sources useful to host [5]. The intestinal microbiota also positively impacts lipid metabolism by enhancing lipid hydrolysis efficiency [6]. The pathogenic burden of the microbiota in sepsis is not fully comprehended. Dietary habits can favor the growth of specific bacterial strains, altering fermentative metabolism and intestinal pH, which may lead to the growth of pathogenic flora [7]. For instance, a diet with an elevated fat content can develop a pro-inflammatory phenotype, increasing intestinal permeability and blood lipopolysaccharide levels [7]. A promising therapeutic option for various diseases resides in manipulating gut balance. However, the efficacy of ongoing treatment strategies, such as FMT, probiotics, and prebiotics, is limited by multiple obstacles. These include the imprecision of treatments, legislative and safety problems, and the difficulty in offering repeatable and targeted procedures [8]. Despite these challenges, the importance of a balanced microbiota for human health is increasingly emphasized (Figure 1).

On the other hand, sepsis is a clinical syndrome caused by an abnormal and multifaceted host defense against an infection, leading to potentially fatal organ dysfunction and influenced by endogenous factors. It is characterized by a systemic inflammatory response and can lead to septic shock and death [9]. Organ impairment is defined as an alteration in the Sequential [Sepsis-related] Organ Failure Assessment (SOFA) score of more than 2 points to infection and carries a 10% in-hospital mortality risk [10].

The actual incidence in emergency departments and general wards remains unknown [11], due to the challenges in collecting large-scale data, particularly in low-income countries [12], as well as the lack of precise and reliable criteria for diagnosing sepsis [10]. Several studies have reported a mortality rate of 26.7% in the group of septic patients treated in hospitals and 41.9% in ICU patients [12]. In 2017, Rudd KE et al. reported a total of 48.9 million cases, with 11 million fatalities worldwide [13]. For a faster bedside diagnosis, qSOFA (quick SOFA) was developed in 2016, incorporating easily measurable clinical criteria:

Glasgow Coma Scale score ≤13, systolic blood pressure ≤100 mmHg, and respiratory rate ≥22 breaths per minute.

In diagnosis, laboratory criteria complement clinical criteria. Among the main biomarkers are C-reactive protein (CRP) and procalcitonin (PCT). Both markers have limitations in sensitivity and specificity, making blood culture in some respects the gold standard, although this method takes a long time. Promising new studies have reported on the role of presepsin as a new useful biomarker for the early diagnosis of sepsis.

In this review, we analyze the dysfunctions of the microbiota before and during sepsis, the impact of antibiotics on immune dysregulation and intestinal microbiota, laboratory markers, and therapeutic targets. The aim of this review is to emphasize the prudent use of antibiotics by highlighting the role of the microbiota in sepsis as well as to collect the existing literature on this topic and therapeutic strategies.

## 2. The Role of Microbiota

**Figure 2:**
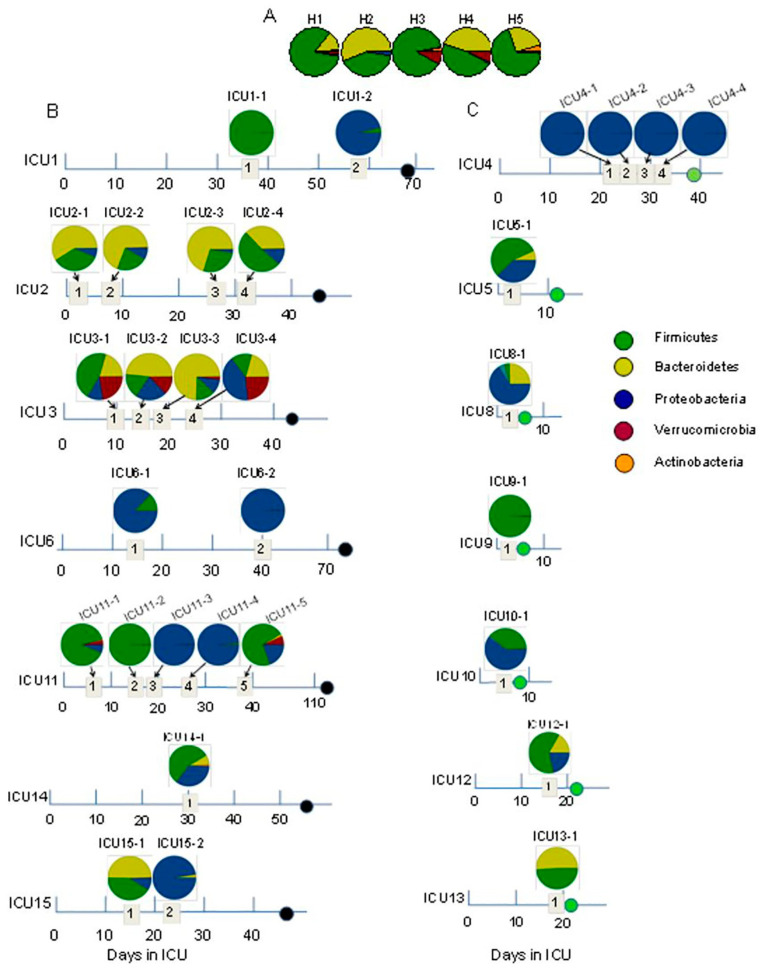
The taxonomic composition of the gut microbiome at the phylum level in healthy volunteers (**A**), ICU patients dying with severe sepsis (as indicated by black circles on the timeline) (**B**), and ICU patients who had recovered (as indicated by green circles on the timeline) (**C**) [16].

The gut microbiome plays a crucial role in resistance to pathogens, even in distant organs such as the lungs. Changes in microbiota in sepsis are responsible for worse prognoses. Reduced microbial diversity and the loss of beneficial bacteria, such as butyrate producers, may be indicators of adverse outcomes [14]. The human gut also hosts eukaryotic viruses, bacteriophages, fungi, archaea, and protozoa, but their role in sepsis remains unclear. Different studies on critically ill patients have demonstrated that the decline in anaerobic bacteria is associated with the growth of aerobic pathobionts and opportunistic yeasts like *Candida* and *Aspergillus* [15] (Figure 2).

The gut microbiota significantly impacts immune function, including local barrier protection, hematopoiesis, T-cell differentiation, cytokine and antibody production, and phagocytosis [17]. As an illustration, *Bacteroides* and *Firmicutes*, the dominant phyla in a healthy gut, produce short-chain fatty acids (SCFAs) such as propionate, acetate, and butyrate, which regulate gene expression in regulatory T cells [18] and enhance the microbicidal capabilities of macrophages by inhibiting histone deacetylase 3 (HDAC3) [19,20]. Thanks to this, there is a reduction in pro-inflammatory cytokines regulated by NF-κB, including TNF-α and IL-6. Additionally, butyrate has been discovered to increase the concentration of interleukin-10 (IL-10), which has reduced inflammatory responses in murine models of septic shock [21]. According to a study by Yamada et al., patients with sepsis exhibit a reduction in fecal SCFAs, which appears to persist for up to 6 weeks [22]. This results in intestinal barrier dysfunction and alters immune response. SCFAs play a crucial role in supporting intestinal epithelial cells by ensuring their proliferation and differentiation [23]. Low SCFA levels represent a negative prognostic factor. Microbiota metabolites modulate key immune pathways. For example, D-lactate, produced by intestinal bacteria and transferred to the liver via the portal vein, is essential for the accuracy of the immune response thanks to Kupffer cells that capture and kill circulating pathogens, preventing the spread to other organs [24]. SCFAs contribute to maintaining intestinal barrier integrity and reducing inflammation. Furthermore, they circulate throughout the human organism using the lymphatic and humoral systems and bind to their specific G-protein-coupled receptors (GPR43, GPR41, and GPR109a), which block the production of inflammatory proteins regulated by histone deacetylase [25]. It follows that SCFA concentrations are closely related to prognosis [26]. Artificial nutrition is essential for septic patients, but dietary composition, particularly rich in animal proteins and fats but low in fiber, can alter the microbiota within a single day, reducing SCFAs and increasing secondary bile acids [27]. However, hospitalized septic patients often receive sterile casein-based diets with no fiber [27]. In intensive care units, there is a risk of patient malnutrition, which holds a negative prognostic value [28]. Early enteral nutrition with a standard formula is generally preferred for nearly all critically ill patients. The addition of parenteral nutrition is assessed on an individual basis [28]. Nevertheless, we still lack sufficient studies to determine the impact of different formulations on prognosis, and conflicting [29,30] evidence exists regarding their effects on the length of stay, inflammatory processes, duration of mechanical ventilation, and mortality. The area of focus should be the potential for enteral nutrition to be enriched with soluble or insoluble fibers derived from soy polysaccharides, partially hydrolyzed guar gum, psyllium, mixed fiber, and pectin to simulate natural, balanced nutrition as closely as possible [31] In patients receiving parenteral nutrition, integrating fiber is more challenging, as these formulations do not contain it. While essential amino acids and polyunsaturated fats can support certain metabolic functions, they cannot replace SCFAs. Huwiler et al. [32] show how the number of studies highlighting the benefits of dietary fibers (DF) in enteral nutrition (EN) for most patients is steadily increasing, due to their positive effects on various mechanisms, such as maintaining mucosal barrier integrity, enhancing cellular defense, and modulating inflammation.

However, current evidence on the impact of DF-enriched EN in septic conditions and related clinical outcomes remains limited and inconclusive. Many concerns persist regarding the high risk of ischemia, severe dysmotility, or susceptibility to food intolerance in ICU patients. A fiber-rich diet can also lead to intestinal distension, increasing the risk of adverse events. Therefore, further large-scale, high-quality clinical trials focusing solely on the effects of dietary fibers, without the influence of other immunonutrients, are needed to achieve clearer conclusions. Immunonutrition could represent another important area of exploration; however, the current evidence is still insufficient. In a multicenter prospective observational study involving 61 treated patients, López-Delgado et al. [33] observed a reduced need for vasopressors and continuous renal replacement therapy, along with improved 28-day survival (85.2% vs. 73.3%, *p* = 0.014).

Further research is needed to develop formulations that can reproduce these short-chain fatty acids, to restore intestinal integrity and improve prognosis in this patient population.

The interaction between the microbiome, epithelium, and immune system regulates gut permeability [34]. The gut microbiota plays a crucial role in maintaining the integrity of the epithelial barrier due to its ability to compete with pathogens and produce metabolites that regulate various host functions. For example, butyrate and propionate stimulate the production of proteins that strengthen intercellular junctions, such as ZO-1 and occludin [35,36], although high doses of butyrate may weaken the barrier by inducing apoptosis [37]. Barrier integrity is maintained by junctional proteins, including claudins, occludins, and cytosolic proteins. It is further strengthened by mesenteric lymph nodes, the lamina propria, Peyer’s patches, and intraepithelial lymphocytes. When this integrity is compromised, apoptosis of the intestinal barrier cells follows. Similarly, polyamines, synthesized by both the microbiota and the host, support barrier integrity by modulating the expression of key proteins [38]. Among other metabolites, conjugated linoleic acid (CLA) exhibits complex effects: while it increases intestinal permeability in vitro, it shows a protective effect in vivo in colitis models [39,40]. In addition to bacterial metabolites, structural components such as lipopolysaccharides and flagellin also influence barrier function by activating specific Toll-like receptors (TLRs), with effects ranging from enhancement to disruption of epithelial permeability [41,42].

Yoseph et al. conducted a study on mice, examining junctional proteins using real-time polymerase chain reaction, Western blot, and immunohistochemistry 12 h after cecal ligation and puncture (CLP), and in a separate group of mice with Pseudomonas aeruginosa infection. In both groups, claudin-2 and JAM-A levels increased with sepsis, while claudin-5 and occludin levels decreased [43].

Intestinal hyperpermeability is mediated by a series of pro-inflammatory cytokines, including TNFα, IL-1β, and IL-6, whose production appears to be influenced by myosin light chain kinase (MLCK). Infections, through the secretion of IL-1β by infected immune cells and the activation of TLR-2, can lead to the upregulation of MLCK [44]. The gut microbiome may regulate this process by influencing the first pathway, namely, competition with pathogens. However, to date, no specific studies have explicitly addressed this phenomenon. Lorentz et al., in a study on mice, demonstrated a survival advantage in sepsis with a knockout of the kinase [45].

Cytokine expression may be influenced by commensal microbes acting on immune pathways. Supporting this hypothesis, a study on 500 healthy adults by Schirmer et al. demonstrated that *Coprococcus comes* influences the production of IL-1β and IL-6 cytokines in response to *Candida albicans* infection [46].

Regarding hematopoiesis, it has been observed that germ-free neonatal mice are more susceptible to sepsis from *Staphylococcus aureus* and *Listeria monocytogenes* due to a reduction in myeloid bone marrow precursors and alterations in the number of splenic macrophages, monocytes, and neutrophils [47].

Furthermore, according to a study by Zhang et al. on antibiotic-treated mice, the microbiome regulates neutrophil aging. In this study, neutrophil extracellular traps were significantly reduced following antibiotic administration [48].

Another study highlighted that the gut microbiome also regulates humoral immunity: commensal bacteria play a role in the production of IgA, which depends on T cells [49].

The impact of symbionts on systemic immunity also appears to be mediated by immunoglobulins. Zeng et al. demonstrated that the systemic production of serum immunoglobulins (Ig) G is induced by antigens expressed on the outer membrane of gram-negative bacteria [50]. Other mediators include bacteriocins, which are extracellular antimicrobial peptides produced by bacteria and archaea from different phylogenetic backgrounds. Bacteriocins offer the capacity to inhibit or eradicate drug-resistant organisms, unlike conventional antibiotics, because they can damage bacterial cell membranes and lead to the loss of intracellular components [51]. Utilizing potent and narrow-spectrum bacteriocins as protein-based antibiotics presents a promising alternative strategy for combating multidrug-resistant bacteria [52].

Several preliminary studies in mice show that the microbiome influences the systemic immune response to illnesses. Intestinal dysbiosis, as demonstrated using germ-free mice or those treated with antibiotics, increases mortality from bacterial infections [53]. Some clinical treatment methods, such as mechanical ventilation, vasoactive drugs, and broad-spectrum antibiotics, can alter intestinal flora and impair its functions [54]. Patients with sepsis exhibit a higher intestinal abundance of *Enterococcus* compared to healthy individuals [54]. This phenomenon appears to be linked to the depletion of SCFA-producing bacteria, which promotes the overgrowth of vancomycin-resistant *Enterococcus* strains in critically ill patients [55]. Specifically, the reduction of butyrate has been associated with an increased presence of these species in the colon [56]. In sepsis, there is often a loss of obligate anaerobes such as *Bacteroidetes* and *Firmicutes*, leading to the proliferation of normally less abundant taxa like *Proteobacteria* (including *E. coli* and *K. pneumoniae*) [57]; additionally, the use of antibiotics promotes colonization by *Clostridium* and vancomycin-resistant *Enterococcus* (VRE) [58]. Patrier et al., in a prospective monocentric cohort study, found that high concentrations of *Enterococcus*, *S. aureus*, and *Candida* were associated with increased mortality, regardless of age, organ failure, and antibiotic therapy [59]. Selective pressures due to physiological stress and treatments (antibiotics, artificial nutrition) influence this alteration [60]. Intensive care unit admission can compromise these defenses, leading to severe complications such as multiple organ failure (MOF), the development of coronary artery disease (CAD), systemic infections, ventilator-associated pneumonia (VAP), healthcare-associated pneumonia (HAP), and acute respiratory distress syndrome (ARDS) [61]. Nevertheless, it is difficult to establish a causal relationship between gut dysbiosis and prognosis in ICU patients, considering that these events directly affect both prognosis and microbiota alteration (Table 1).

## 3. The Impact of Antibiotics on Microbiota

A common alteration of a healthy microbiota is due to the use of antibiotics. These drugs, especially those with anti-anaerobic activity, can drastically modify microbial ecology, favoring the predominance of normally exiguous but highly pathogenic species such as *Enterococcus faecium* and *Klebsiella pneumoniae* [63]. Exposure to broad-spectrum antibiotics in neonatal mice reduces type-3 innate lymphoid cells and increases susceptibility to sepsis [64]. Two large retrospective studies have further validated the link between microbiota impairment and sepsis exposure, finding that patients who were exposed to a high likelihood of dysbiosis or received a higher amount of antibiotics during their hospital stay had an increased risk of sepsis within 90 days of discharge [65]. Among 10,996 patients, the incidence of rehospitalization for critical sepsis was 70% higher after *Clostridium difficile* infection compared to other infections [65]. In animal models of sepsis, antibiotic pretreatment or germ-free conditions can prevent lung damage [66]. One study also highlighted the association of colonization with *Klebsiella pneumoniae* to sepsis. While the administration of *Lactobacillus murinus* offers protection [67], this confirms the significant impact of the microbiota on the onset, progression, and prognosis of sepsis. Antibiotics influence the gut microbiome beyond their spectrum of activity. For example, vancomycin reduces *Bacteroidetes*, while metronidazole increases the risk of *Enterococcus* dominance compared to other antibiotics [60]. Research has demonstrated that frequent use of third-generation cephalosporins raises the probability of getting colonies of *Enterobacteriaceae* and, less markedly, multi-drug-resistant gram-positive bacteria [68]. Clindamycin, primarily eliminated through the biliary routes, reaches high concentrations in the feces and causes significant alterations in the gut microbiota, reducing anaerobes and slightly increasing Gram-positives and enterobacteria. This drug also induces dysbiosis that favors multi-drug-resistant pathogens and increases the risk of *Clostridium difficile* colitis [69]. The use of amoxicillin, alone or in combination with a beta-lactamase inhibitor, is related to a decline in *Lactobacillus* spp. and a growth in multi-drug-resistant *Enterobacteriaceae*, with changes in the gut microbiome lasting up to two months [70]. Intestinal dominance of *Enterococcus* has been found to be associated with the risk of death in septic patients in intensive care; no causal relationship has been demonstrated, but *Enterococcus* can lead to different alterations [71]. These alterations can reduce SCFAs, promote antibiotic resistance, and increase the expression of virulence factors [16,72]. In this review, carbapenems are not mentioned, despite being among the most widely used antibiotics, as no studies within our analysis timeframe were identified. The recovery of the microbiota after antibiotic use can take weeks or months, and in some cases up to a year [73]. The absence of lactose in the diet has been observed to reduce the growth of *Enterococcus*, suggesting a potential therapeutic strategy [74].

In septic patients with interrupted nutrition, there is increased administration of antibiotics and greater dysbiosis, with a loss of anaerobes, reduction in SCFAs, and an increase in pathogens, associated with bacteremia, organ failure, and death [75]. A study showed the emergence of a virulent, multi-drug-resistant pathobiome in prolonged critical patients, with gut communities dominated by single antibiotic-resistant pathogens [16] (Table 2).

## 4. Microbiota as a Predictive Indicator of Sepsis

**Figure 3:**
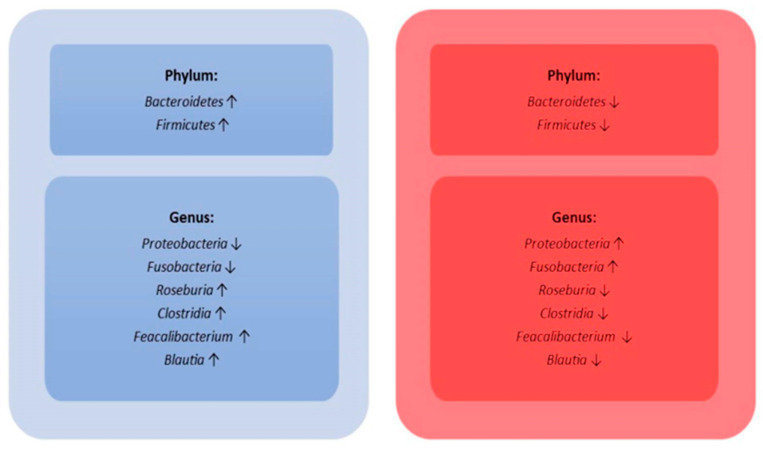
Gut microbiota variability according to healthy status (light-blue box) and during sepsis (red box) [77].

The gut microbiota has the potential to serve as a biomarker for identifying patients at higher risk of developing sepsis. However, while some studies have demonstrated associations between specific microbial patterns and the onset of sepsis, these findings need to be interpreted cautiously, as reproducibility across different patient populations and settings has not yet been consistently established. Moreover, it is crucial to emphasize that association does not imply causation, and the current evidence is insufficient to support the immediate use of microbiota profiles as reliable biomarkers in clinical practice. This area of research shows promise, but further robust and large-scale studies are required to validate these associations and determine their clinical applicability in predicting sepsis risk. Therefore, while the microbiota might represent a future avenue for investigation, it is not yet ready to be used as a definitive biomarker in sepsis management. One study found that the presence of fecal pathogens before sepsis is a predictive indicator. The causal bacterium of gram-negative newborn sepsis was identifiable in at least one of the stool samples collected three days before the onset of sepsis in all cases, although this pathogen was undetectable in all matched controls [76] (Figure 3).

Most cultured pathogens were CoNS (67.5% Staphylococcus epidermidis), followed by other gram-positive bacteria such as Enterococcus faecalis and Staphylococcus aureus, followed by gram-negative bacteria such as Escherichia coli and Klebsiella pneumonia [76]. In a prospective cohort of 71 preterm newborn with late-onset sepsis, Bacilli (mostly CoNS staphylococci) characterized the gut microbiota, and the quantity of anaerobic bacteria (for instance Clostridia) was reduced prior to sepsis onset [78].

An innovative study on 708 adults showed that intestinal dominance by Proteobacteria was linked to a seven-fold higher risk of gram-negative bloodstream infection later on [79]. The results of these studies derive from a small sample size, and the authors themselves have emphasized the need for larger studies to ensure the reproducibility of their conclusions. The reproducibility of these findings remains a major concern due to differences in study designs, patient characteristics, and microbiota analysis techniques. It is important to highlight that these studies primarily show associations rather than a direct cause-and-effect relationship. It is unclear whether the observed microbial changes are the cause of sepsis or merely a consequence of other underlying conditions. Further studies focused on the adult population are urgently needed since few studies have confirmed these results in this population. Using 131 stool samples from 64 critically ill patients suffering from sepsis or septic shock, it was finally observed that these Chinese patients, despite having a variety of illness types and receiving different antibiotics, consistently displayed one of two microbiota patterns (enterotypes).

Elevated serum lactate levels were linked to the first enterotype, known as ICU E1, which contained Bacteroides and a major unclassified genus of Enterobacteriaceae. On the other hand, Enterococcus predominated in ICU E2, and Bacteroides was lost [80].

Gaining a better comprehension of the molecular processes that lead to sepsis is essential for timely diagnosis and the development of effective treatment plans.

Research is currently underway to assess the gut microbiota as an indicator to predict therapeutic response thanks to NGS sequencing in conjunction with artificial intelligence [81]. It is important, therefore, to develop new techniques for obtaining early reports on microbiology. These could include nucleic acid amplification technologies (NAATs) that amplify nucleic acid sequences and identify the infectious agent or immune response status. The detection of bacterial DNA fragments by real-time polymerase chain reaction (RT-PCR) in blood samples and the detection of 16S rRNA fragments of Gram-positive and gram-negative bacteria or 18S rRNA fragments of *Candida* spp. seem to have a great potential for shortening pathogen identification, as they have demonstrated high levels of sensitivity, which could reduce patient mortality, hospital stay duration, and ICU stays [77]. Therefore, there are no biomarkers that can exclusively recognize septic individuals, and these methods are insufficient to distinguish sepsis from other inflammatory conditions (Table 3).

## 5. Biomarkers of Intestinal Dysbiosis

The gut microbiota plays a crucial role in maintaining the integrity of the intestinal barrier [82]. A diverse and balanced microbiota strengthens tight junctions between intestinal epithelial cells, which reduces permeability and prevents harmful substances from translocating into the bloodstream [83]. Dysbiosis, or an imbalance in the microbial community, allows for the translocation of bacteria, endotoxins (such as lipopolysaccharides), and other microbial products into systemic circulation. The overgrowth of pathogenic bacteria can disrupt the epithelial barrier, leading to an increased translocation risk. Factors such as antibiotic use, diet, and underlying diseases can further influence gut microbiota composition and functionality, thereby affecting the risk of translocation. For example, antibiotics can disrupt the microbiota balance, promoting the growth of pathogenic organisms that compromise the intestinal barrier [84]. Endothelial dysfunction is associated with poor outcomes in critically ill patients, including those with sepsis [85]. The link between gut microbiota, translocation, and endothelial dysfunction suggests that alterations in microbiota may indirectly influence prognosis through their impact on endothelial health. For instance, microbial translocation can activate inflammatory pathways that contribute to endothelial injury [86]. However, the direct relationship between microbiota composition and endothelial dysfunction is still under investigation. To evaluate these alterations in the microbiota, various markers are available. Measurements of LPS, citrulline, the lactulose test, FABP, and fecal calprotectin are emerging as excellent alternatives with high specificity and sensitivity. Citrullinemia testing is also applicable in clinical settings to assess enterocyte functionality in critical patients, as it is relatively easy to administer [87]. Citrulline is a non-protein amino acid produced by enterocytes in the small intestine and is used as a marker of intestinal function. Citrulline levels decrease in critical illnesses and sepsis due to the depletion of nitric oxide and arginine in inflammatory pathways [88]. However, they increase once the critical condition is overcome, acting as a negative inflammatory marker. It is unclear if citrulline values represent gut function (particularly absorption), the mass of enterocytes, a mixture, or other factors [89]. Intestinal fatty acid-binding protein (I-FABP) and diamine oxidase (DAO) are cytosolic proteins in intestinal epithelial cells, released rapidly into the bloodstream when the intestinal barrier is damaged [90]. Diamine oxidase (DAO), also known as histamine oxidase, is found in a variety of tissues, with substantial expression in the mucosa of the small intestine [91]. DAO is an enzyme mainly formed by absorptive cells at the tips of small intestine villi, with activity increasing from the duodenum to the ileum. Low DAO levels in the blood indicate the maturity and integrity of the intestinal mucosa, making it a reliable measure for monitoring mucosal function. Small amounts of DAO enter the systemic circulation, serving as a marker for the quantity of mature and functioning enterocytes [92]. Conversely, during intestinal ischemia and other multiorgan dysfunction syndromes, enterocytes intensely release DAO into the blood [92]. Intestinal fatty acid-binding protein (I-FABP) is present in mature epithelial cells of the small intestine and acts as a marker of epithelial cell integrity: when the intestinal mucosa is injured or compromised, I-FABP is released into the blood, increasing its concentration. In regular circumstances, I-FABP content in tissues is elevated, while it stays low in serum [91]. DAO and I-FABP directly indicate different aspects of intestinal epithelial barrier cell damage, offering a quantitative and qualitative assessment of intestinal barrier function. Thus, in a septic patient, DAO and I-FABP will be increased. In a study with twelve rats, Eva Lau et al. observed that animals fed high-fat (HF) diets develop obesity, insulin resistance, and show increased plasma levels of pro-inflammatory cytokines (MCP-1 and IL1β). Other indicators of bacterial translocation due to intestinal barrier disruption include Endotoxin (ET), specific to gram-negative bacteria. When barrier function is compromised, significant amounts of ET enter the bloodstream, leading to an imbalance of ET levels in the blood [93].

Lipopolysaccharide (LPS) and presepsin are used as biomarkers of bacterial translocation. LPS, a component of the gram-negative bacterial wall, acts as a direct biomarker of bacterial translocation, while presepsin, derived from CD14 protein, is released after bacterial phagocytosis [92]. Presepsin can indicate bacterial translocation in the absence of obvious infection sources, serving as an indirect marker for both Gram-positive and gram-negative bacteria [92].

Finally, the lactulose hydrogen breath test, used for diagnosing SIBO, as recommended by the North American Consensus and the national scientific organization [94], might find an application in evaluating intestinal barrier dysfunction.

## 6. Therapeutic Opportunities

Various strategies are being developed to modulate the gut microbiota, such as selective digestive decontamination (SDD), the use of probiotics, prebiotics, symbiotics, postbiotics, and fecal microbiota transplantation (FMT).

SDD reduces respiratory infections and mortality, but concerns about antibiotic resistance remain. Its effect on bloodstream infections (BSI) was not as pronounced, and the mechanism behind this discrepancy remains unclear.

One explanation could be that SDD primarily targets the gut microbiota, reducing the burden of potential respiratory pathogens, but may not completely eliminate the translocation of bacteria into the bloodstream. This may be due to selective resistance patterns [95], the presence of undetected translocating pathogens, or incomplete decontamination.

SDD is a preventive measure involving the use of non-absorbable topical antimicrobials to preserve the anaerobic microbiota of the upper respiratory and gastrointestinal tract. This strategy can be applied alone or with a short course of broad-spectrum antibiotics administered intravenously, aiming to decrease or prevent endogenous infections. SDD has not shown an increase in the prevalence of antibiotic resistance and might even be associated with a lower acquisition of resistant bacteria. In contrast, SDD has been linked to the eradication and reduced acquisition of rectal third-generation cephalosporin and carbapenem-resistant gram-negative bacteria among mechanically ventilated patients in a randomized cross-over study [96]. Further research is needed to elucidate the exact mechanisms and optimize SDD protocols to reduce the risk of BSI without promoting antimicrobial resistance.

Other interventions to modify the microbiota include probiotics, prebiotics, symbiotics, and postbiotics, which may prevent sepsis and improve patient prognosis. Probiotics are living microorganisms that, when consumed in sufficient quantities, can provide health benefits to the host [97]. They have also been employed in neonatal sepsis and necrotizing enterocolitis. Current research is still insufficient and shows mixed results. A meta-analysis revealed how probiotic consumption reduces the hazard of late-onset sepsis, from 16.3% in the placebo group to 13.9% in the probiotic group [98]. Generally, probiotics are well-tolerated. Probiotics vie with native bacteria for essential nutrients and attachment points, generate bacteriocins to target harmful pathogens, boost IgA levels, strengthen mucosal defenses, and help diminish overall inflammation in the body [99]. Another investigation found that administering probiotics led to an increase in bacterial translocation among patients experiencing organ failure [100], highlighting potential risks of bacteremia associated with their administration. Probiotics were also evaluated in a large multicenter study, the results of which indicated that they do not reduce the risk of ventilator-associated pneumonia or other sepsis-related outcomes in intensive care units [101].

Prebiotics are substrates that support the growth and activity of beneficial microorganisms in the host, leading to positive health effects [102]. They are naturally found in foods such as milk, cereals, asparagus, onions, garlic, and vegetables. The most common types of prebiotics include fructooligosaccharides, galactooligosaccharides, and trans-galactooligosaccharides [103]. Prebiotics promote an increase in beneficial species (Akkermansia, Terrisporobacter, and Anaerostipes), and stimulated acetic and propionic acid production [104]. There is insufficient evidence to confirm a direct clinical benefit in sepsis prevention or treatment. Most studies remain preclinical or observational, lacking the robust data needed to support their routine use in critical care settings.

Symbiotics are formulations that blend live microorganisms with substances that can be utilized by native and non-native host microorganisms [105]. A meta-analysis supports their efficacy in reducing septic complications in critically ill patients, but these findings were not statistically significant [106]. The heterogeneity of patient populations and the variability in symbiotic formulations limit the generalizability of these results. Overall, the existing data suggest that while probiotics, prebiotics, and symbiotic can modulate gut microbiota and reduce certain markers of inflammation, their impact on sepsis outcomes remains inconclusive.

**Figure 4:**
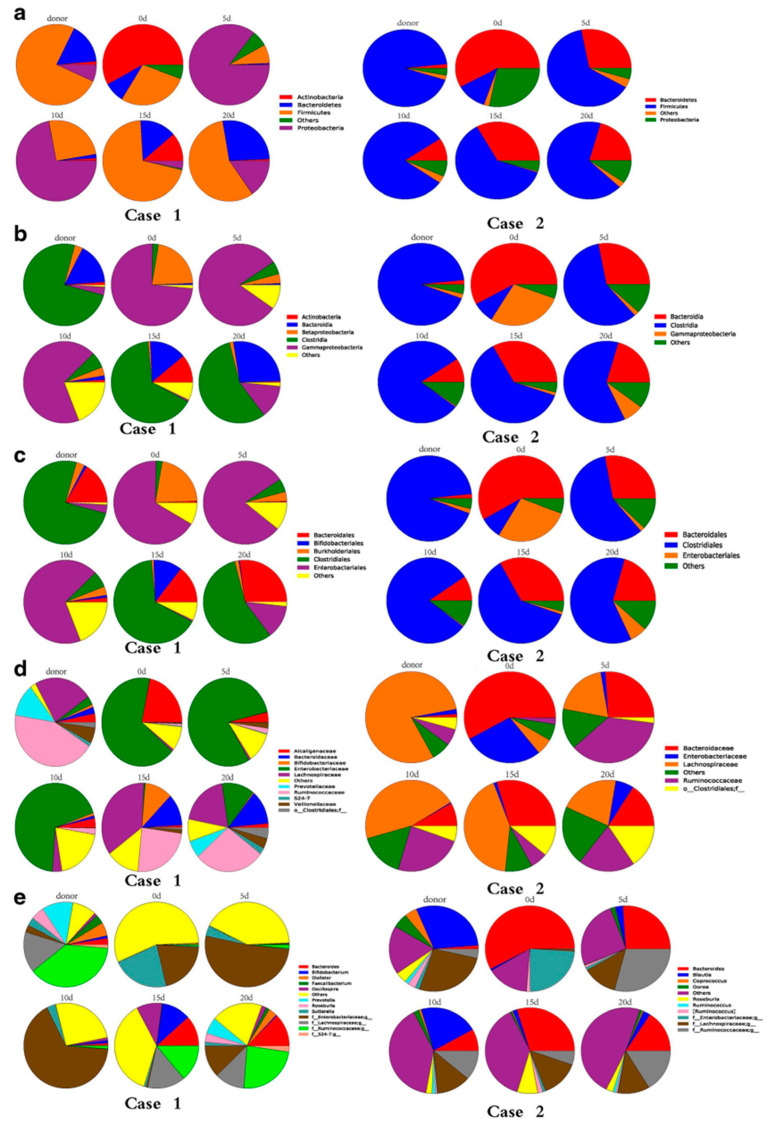
Predominant fecal microbiota composition in the donor and patients (case 1 and case 2) at the phyla level (**a**), the class level (**b**), the order level (**c**), the family level (**d**), and the genus level (**e**). Variations in the microbiota composition are shown at the representative time points in the days following fecal microbiota transplantation [111].

According to the International Scientific Association of Probiotics and Prebiotics, postbiotics are a “preparation of inanimate microorganisms and/or their components that confers a health benefit on the host” [107]. Postbiotic components are divided into two main groups. The first includes elements from beneficial bacteria such as lipoic acid, phosphonic acid, peptidoglycans, cell surface proteins, polysaccharides, membrane proteins, and extracellular polysaccharides. The second group comprises metabolites from beneficial bacteria, such as vitamins, lipids (butyrate, propionate, acetate, lactic acid, etc.), enzymes, proteins (p40, p75 molecules), peptides, organic acids (propionic acid, 3-phenyllactic acid, etc.), SCFAs, and intracellular polysaccharides [108]. The diversity of these postbiotic components results in numerous functions, including antibacterial activity, immune system regulation, antioxidant activity, liver protection, blood pressure reduction, gut flora regulation, and the prevention and treatment of constipation, enteritis, and other conditions [109]. Postbiotics are generally safer for vulnerable groups, such as infants and sensitive individuals, compared to probiotics, which can carry risks and negatively interact with antibiotics. Additionally, postbiotics are more stable and resistant to environmental conditions like oxygen and temperature, offering a superior shelf life compared to live probiotics [110]. Despite the different supporting studies, we still need to confirm the effectiveness of prebiotics/probiotics/symbiotics in the prevention and treatment of different diseases [104] (Figure 4).

Finally, FMT, by transferring bacteria and other microorganisms, seems to offer advantages over other microbiota-targeted therapies. The methods of administering fecal microbiota transplantation (FMT) include oral capsules, nasojejunal and nasoduodenal tubes for the upper gastrointestinal tract, and colonoscopies and enemas for the lower gastrointestinal tract [112]. Careful donor selection is crucial to prevent the transmission of pathogens. The success of FMT depends on the ability to correct dysbiosis by restoring bacteria such as Roseburia and Bacteroidetes, which are essential for butyrate production [112]. Microbiota-targeted therapies, such as FMT or SCFAs, have demonstrated potential in preventing acute kidney injury [113,114], though their relevance to humans is still to be established [115]. FMT, effective against Clostridioides difficile infection, has been experimented with in cases of sepsis but requires further studies to verify its efficacy [111]. In a randomized clinical trial, it was observed that multiple fecal infusions combined with vancomycin were more effective than a single transplant in treating Clostridium difficile infection [116]. However, a recent case showed the transmission of a multidrug-resistant organism via FMT, causing lethal bacteremia in two patients [117], urging the need for extreme caution and screening in using FMT [118] (Table 4).

## 7. Materials and Methods

For the following narrative review, the materials were retrieved through the PubMed electronic database. Approximately one hundred studies were considered to understand the interaction between sepsis and microbiota in pathogenic, diagnostic, and therapeutic contexts. The articles were identified through a comprehensive search combining key terms such as “intestinal microbiota”, “sepsis and microbiota”, “prebiotics, probiotics, postbiotics, symbiotics, and intestinal disease”, “antibiotics and microbiota”, “diet and microbiota”, “microbiota biomarkers”, and “citrulline and microbiota”. Additionally, the reference lists of the chosen articles were reviewed to find other pertinent studies. Only English-language articles published in the last 15 years were included (54.6% of the analyzed works were published between 2019 and 2024).

## 8. Conclusions

Evidence suggests that the microbiome has a crucial influence on the progression and outcome of sepsis by contributing to immune dysregulation, which results in organ failure. Alterations of microbiota have been linked to a higher susceptibility to sepsis and an amplified hazard of negative outcomes. Recently described processes highlight how the dialogue between microbiota-derived metabolites and immune cells can influence the pathogenesis of sepsis. However, much of this evidence is based on correlation or preclinical studies and has yet to be confirmed clinically. The causal relationship between microbiota, metabolic or immune dysregulation, sepsis onset, and prognosis is not established. In addition, the impact of non-bacterial gut inhabitants on sepsis remains to be elucidated.

In the future, microbiota-targeted therapies could play a crucial role in guiding an immune response oriented towards recovery. While promising, microbiome-targeted therapies remain largely experimental at present. Currently, the judicious use of antibiotics and a reconsideration of existing nutritional formulations are the only recommended therapeutic treatment based on current evidence. The availability of few prognostic and therapeutic tools based on the microbiota limits clinical practice. The advancement of rapid and straightforward microbiota-targeted assays has the potential to enhance risk assessment and enterotype classification in the context of sepsis. A bottom-up approach to identify patients who would benefit most from microbiota-targeted therapies could make such therapies safer and more advantageous. Ultimately, this could lead to a more personalized approach in managing sepsis in the years to come.

## Figures and Tables

**Table 1:** Works supporting “The role of microbiota”.

Authors	Type	Year	Subjects	Findings
Arpaia et al. [18]	Single-center case-control study	2021	Five mice in each group. Pathogen-free mice (SPF), others treated with broad-spectrum antibiotics (AVNM), and still others germ-free (GF).	After the administration of butyrate, an increase in extrathymic Treg cell levels was observed.
Schulthess et al. [19]	Observational study	2019	Intestinal macrophages in vivo.	Butyrate induced a reduction in mTOR kinase activity and the production of antimicrobial peptides without an increased inflammatory cytokine response. Butyrate drove the differentiation from monocytes to macrophages through the inhibition of histone deacetylase 3 (HDAC3).
Wang et al. [21]	Single-center case-control study	2017	Male mice were randomly divided into the following groups: septic model group (M), normal control group (NC), and SCFA pretreatment groups.	Butyrate, a short-chain fatty acid (SCFA), significantly reduced inflammation in response to sepsis by enhancing the expression of the anti-inflammatory cytokine IL-10 (*p* < 0.01).
Yamada et al. [22]	Single-center case-control study	2015	140 ICU patients with SIRS criteria and PCR level >10 mg/dL. Fecal samples were used for the quantitative measurement of SCFA concentrations.	The levels of butyrate, propionate, and acetate in the feces of these patients were significantly lower than those in healthy volunteers and stayed low throughout the entire 6-week ICU stay.
McDonald et al. [24]	Single-center case-control study	2020	Pathogen-free (SPF) and germ-free (GF) mice were infected with Staphylococcus aureus via intravenous injection.	The gut microbiota supports the removal of circulating pathogens by Kupffer cells in vivo through D-lactate produced by commensal bacteria, which travels to the liver through the portal vein (*p* < 0.05).
Wang et al. [26]	Single-center case-control study	2024	Thirty-six healthy, 8-week-old male C57BL/6J mice, maintained in pathogen-free conditions, were randomly assigned to four groups: Control, LPS, EcN, and EcN + LPS.	Pretreatment with Escherichia coli Nissle (EcN) can significantly increase the abundance of Bacteroidetes (produce high levels of acetate) and Firmicutes (significant amounts of butyrate) in mice with septic shock. This intervention not only enhances intestinal barrier function but also positively modulates gut microbiota composition.
Grillo-Ardila et al. [29]	Meta-analysis	2024	Five RCTs (n = 442 participants) and ten NRSs (n = 3724 participants) were included.	Limited evidence indicates that Exclusive Enteral Nutrition (EEN) may be a safe and potentially effective intervention for supporting gut microbiota in critically ill patients with sepsis or septic shock.
Kaewdech et al. [31]	Meta-analysis	2022	Fiber supplementation for hospitalized adults on enteral nutrition was reviewed, including 16 randomized controlled trials (RCTs) from a total of 4469 studies found.	Fiber supplements help alleviate post-meal diarrhea in hospitalized patients who are receiving enteral nutrition (*p* = 0.005). This is likely due to the production of SCFAs following bacterial metabolism.
Lopez-Delgado et al. [33]	Multicenter-observational study	2022	406 patients were included in the analysis, of whom 61 received IMN.	Patients treated with IMN formulas received a higher mean caloric and protein intake, and better 28-day survival rates (*p* < 0.001).
Saleri et al. [36]	Preclinical study	2022		Acetate stimulated cell viability and NO production in a dose-dependent manner (*p* < 0.05), activating a barrier response through claudin-4 and immunity via β-defensin 1 (*p* < 0.05). Propionate supplementation showed similar effects on these parameters. Additionally, SCFA supplementation significantly induced β-defensin 1 expression (*p* < 0.05).
Zhan et al. [41]	Single-center case-control study	2022	Twenty wild-type and ten TLR4 knockout (KO) mice were used to establish a sepsis-induced dysfunctional intestinal barrier model through intraperitoneal injection of lipopolysaccharide (LPS, 10 mg/kg).	The deficiency of TLR4 mitigated LPS-induced intestinal barrier dysfunction by reducing inflammatory responses (*p* < 0.01) and apoptosis (*p* < 0.01), preventing intestinal damage, and modulating gut microbiota dysbiosis.
Gu et al. [42]	Preclinical study	2016		TLR2 signaling in intestinal epithelial cells can enhance barrier function and prevent DON-induced epithelial barrier dysfunction.
Yoseph et al. [43]	Randomized controlled trial	2017	Male and female FVB/N mice aged between six and twelve weeks. Randomized to undergo cecal ligation and puncture (CLP) or sham laparotomy.	Claudin-2 and JAM-A increased in sepsis, while claudin-5 and occludin decreased in response to sepsis (*p* < 0.005). In this case, the disruption of the intestinal barrier could be associated with the gut microbiota; however, there is also a component linked to pro-apoptotic stimuli in the intestinal epithelium due to mitochondrial dysfunction caused by sepsis [62].
Jung et al. [44]	Single-center case-control study	2012	Four groups of mice were used: WT (Wild-Type) as a control, Tlr2^−/−^ mice, Tlr4^−/−^ mice, and Myd88^−/−^ mice.	Upon TLR-2 stimulation, Y. pseudotuberculosis-infected monocytes activated caspase-1 and produced IL-1β. Subsequently, IL-1β enhanced NF-κB activation and myosin light chain kinase (MLCK) expression in intestinal epithelial cells, thereby disrupting the intestinal barrier by opening tight junctions.
Lorentz et al. [45]	Single-center case-control study	2017	Male and female mice, aged six to twelve weeks, with a genetic deletion of the long MLCK isoform, as well as wild-type (WT) mice.	Improved intestinal barrier function in MLCK^−/−^ mice was associated with increased levels of the tight junction mediators ZO-1 and claudin-15. Survival was significantly increased in MLCK^−/−^ mice (*p* < 0.0001). Infections can lead to the upregulation of MLCK, so the gut microbiome may regulate this process through competition with pathogens.
Schirmer et al. [46]	Single-center cohort study	2016	Fecal samples from 500 healthy individuals were collected to generate microbial taxonomic and functional profiles, along with simultaneous blood samples to assess cytokine responses.	*Coprococcus comes* showed a specific association with IL-1β and IL-6 in response to *C. albicans* hyphae stimulation. Furthermore, *C. comes* was inversely related to IL-22 production triggered by *S. aureus*.
Khosravi et al. [47]	Single-center case-control study	2014	Pathogen-free (SPF) and germ-free (GF) mice were infected with Listeria monocytogenes.	Germ-free mice lack myeloid cell populations in the spleen and bone marrow. The microbiota supports the restoration of myelopoiesis and enhances early resistance to systemic infection by Listeria monocytogenes (*p* < 0.05).
Zhang et al. [48]	Single-center case-control study	2015	Neutrophil populations in germ-free (GF) mice compared to specific pathogen-free (SPF) animals.	The microbiota influences neutrophil aging via Toll-like receptor (TLR) signaling pathways and myeloid differentiation factor 88 (MyD88).
Wilmore et al. [49]	Single-center case-control study	2018	C57BL/6 (B6) mice raised in PENN-SPF conditions compared to age-matched JAX-SPF B6 mice.	An increase in Proteobacteria in the microbiota led to IgA-mediated resistance to polymicrobial sepsis. Commensal microbes directly affect the serum IgA profile.
Zeng et al. [50]	Single-center case-control study	2016	Naive wild-type (WT) mice that are either specific pathogen-free (SPF) or germ-free (GF), compared to J H^−/−^ SPF mice with immunoglobulin and B cell deficiencies.	Symbiotic gram-negative bacteria induce an immunoglobulin G (IgG) response against gram-negative bacterial antigens, which provides protection against systemic infections by E. coli and Salmonella. T cells and Toll-like receptor 4 on B cells play a crucial role in generating microbiota-specific IgG.
Schuijt et al. [53]	Single-center case-control study	2016	C57BL/6 mice with depleted microbiota were subsequently infected intranasally with *S. pneumoniae* and then subjected to fecal microbiota transplantation (FMT).	Fecal microbiota transplantation (FMT) in mice with an impaired gut microbiota restored normal lung bacterial counts and levels of tumor necrosis factor-alpha (TNF-α) and interleukin-10 (IL-10) six hours after pneumococcal infection. Whole-genome analysis of alveolar macrophages showed that metabolic pathways were upregulated without a healthy gut microbiota (*p* < 0.05).
Lou et al. [54]	Single-center case-control study	2023	16S rRNA sequencing of fecal samples from both healthy individuals and sepsis patients was conducted to explore whether alterations in gut bacteria are linked to sepsis. A mouse sepsis model was created using cecal ligation and puncture (CLP) to investigate the impact of fecal microbiota transplantation (FMT) and short-chain fatty acids (SCFAs).	Mice with gut microbiota disturbances (ANC group) exhibited a higher risk of death, inflammation, and organ failure compared to mice subjected to CLP (*p* < 0.05).
Livanos et al. [55]	Single-center case-control study	2018	93 patients in intensive care were evaluated 72 h after admission.	A significant decrease in the proportion of Clostridial Clusters IV/XIVa, taxa that produce short-chain fatty acids (SCFA), was observed. At the same time, a significant expansion of Enterococcus was noted, associated with antibiotic use (*p* < 0.01).
Ubeda et al. [58]	Single-center case-control study	2010	Twelve mice were treated with antibiotics to assess changes in the microbiota.	In patients undergoing allogeneic hematopoietic stem-cell transplantation, intestinal dominance by vancomycin-resistant enterococci (VRE) often preceded bloodstream infection (*p* < 0.001).
Patrier et al. [59]	Single-center cohort study	2022	A total of 95 patients were included, with 765 oropharyngeal and rectal samples.	Oropharyngeal and rectal concentrations of Enterococcus spp., Staphylococcus aureus, and Candida spp. were associated with a higher risk of death. This association remained significant after adjustment for prognostic covariates (age, chronic illness, daily use of antimicrobial agents, and daily SOFA score).
Hayakawa et al. [60]	Single-center case-control study	2011	Fifteen patients who suffered a sudden and severe event, along with 12 healthy volunteers as a control group, had fecal samples collected using rectal swabs within 6 h of their emergency room arrival.	Obligate anaerobes and Lactobacillus significantly decreased, and the levels of major short-chain fatty acids in patients were notably lower than those in the control group. The gut microbiota and concentrations of key short-chain fatty acids did not return to normal levels. Conversely, Enterococcus and Pseudomonas increased over the study period.
Taur et al. [63]	Dominant organisms typically included Enterococcus, Streptococcus, and various Proteobacteria. Metronidazole treatment led to a threefold increase in Enterococcus dominance, whereas fluoroquinolone treatment resulted in a tenfold reduction in Proteobacteria dominance.	2012	Fecal samples were gathered from 94 patients receiving allogeneic hematopoietic stem-cell transplantation (HSCT) at various times, from before the transplant through to 35 days post-transplant.	Dominant organisms typically included Enterococcus, Streptococcus, and various Proteobacteria. Metronidazole treatment led to a threefold increase in Enterococcus dominance, whereas fluoroquinolone treatment resulted in a tenfold reduction in Proteobacteria dominance.

SPF = specific pathogen-free mice, AVNM = broad-spectrum antibiotics, GF = germ-free, HDAC3 = inhibition of histone deacetylase 3, SCFA = short-chain fatty acid, M = model group, NC = normal control, EcN = Escherichia coli Nissle, LPS = Lipopolysaccharide, EEN = Exclusive enteral nutrition, KO = knock out ICU = intensive care unit, SIRS = systemic inflammatory response syndrome, PCR = C-reactive protein, DON = Deoxynivalenol, RCT = randomized controlled trials, CLP = cecal ligation and puncture, WT = wild-type, MLCK = myosin light chain kinase, FMT = fecal microbiota transplantation, VRE = vancomycin-resistant enterococci, HSCT = hematopoietic stem-cell transplantation.

**Table 2:** Enhancing the clarity and presentation of “the impact of antibiotics on microbiota”.

Authors	Type	Year	Antibiotics	Findings	Subjects
Taur et al. [63]	Single-center cohort study	2012	Metronidazole fluoroquinolones	3-fold increase in the risk of enterococcal domination. 10-fold decrease in the risk of proteobacterial domination.	94 patients
Niu et al. [64]	Single-center case-control study	2020	Empirical broad-spectrum antibiotics	Expansion of Proteobacteria (*p* < 0.01) Translocation of *E. coli* into the liver and spleen with increased susceptibility to sepsis from *K. pneumoniae*. Decrease in type 3 innate lymphoid cells (ILC3).	
Singer et al. [67]	Single-center cohort study	2019	Gentamicin Vancomycin	Relative abundance of Rodentibacter and Lactobacillus deficiency. Rodentibacter deficiency and normal presence of Lactobacillus.	
De Lastours et al. [68]	Single-center case-control study	2018	Ceftriaxone	Colonization of AmpC-producing Enterobacteriaceae (*p* = 0.02).	15 ceftriaxone and 22 control patients
Smits et al. [69]	Review	2016	Clindamycin, cephalosporins, fluoroquinolones	Increase the risk of Clostridium difficile infection and development of MDR pathogens.	
Zimmerman et al. [70]	Systematic review	2019	Cephalosporins, macrolides, clindamycin, amoxicillin, amoxcillin/clavulanate quinolones, lipopolyglycopeptides, ketolides, tigecycline, and fosfomycin. Cephalosporins, sulfonamides, macrolides, amoxcillin, clindamycin, quinolones Quinolones, piperacillin, macrolides, carbapenems, clindamycin Cephalosporins (except fifth-generation cephalosporins), Amoxicllin, carbapenems, piperacillin and ticarcillin, lipoglycopeptides Doxycycline and macrolides Amoxcillin/clavulant	Increase in abundance of Enterobacteriaea other than *E. coli*, such as *Enterobacter* spp. *Klebsiella* spp. and *Citrobacter* spp. *E. coli* deficiency. Deficiency of anaerobic bacteria. Increased abundance of *Enterococcus* spp. Enterecoccus deficiency. Increased *E. coli*.	2076 participants and 301 controls
Zhao et al. [72]	Single-center case-control study	2016	Cefdinir Azithromycin	Reduces the levels of acetic acid, propionic acid, and butyric acid. After the end of dosing, the levels of butyric acid and valeric acid remained low (*p* < 0.01). Reduces the concentrations of all SCFAs (except hexanoic acid). The gut microbiota recovered, but did not reach the normal level within 8 days of stopping azithromycin (*p* < 0.05).	18 rats, randomly divided into three groups, two experimental groups and a control group

**Table 3:** Works supporting “microbiota as a predictive indicator of sepsis”.

Authors	Type	Year	Subjects	Findings
El Manouni El Hassani et al. [76]	Longitudinal, multicenter, case-control study	2021	There were forty LOS cases (preterm infants born under 30 weeks of gestation) and forty matched controls.	The causing pathogen in gram-negative LOS was found in at least one of the stool samples that were taken three days before the start of the illness. Gram-negative and gram-positive LOS (except CoNS) combined had at least 1 stool sample taken three days before the start of LOS that contained the causal patogen in 92% of the fecal samples. In general, it was possible to forecast LOS (expect CoNS) one day before clinical start.
Graspeuntner et al. [78]	Single-center cohort study	2019	Faecal samples from 164 unaffected controls and 71 premature newborns with LOS.	Anaerobic bacteria are decreased and Bacilli and their fermentation products accumulate during the intestinal dysbiosis that precedes LOS.
Stoma et al. [79]	Retrospective, observational study	2021	708 allogeneic hematopoietic cell transplant (allo-HCT) subjects were studied with 4768 fecal samples for analysis.	In the context of allo-HCT, gram-negative intestinal colonization is a strong predictor of BSI. Fluoroquinolones seem to affect gut colonization, and suppress these infections.
Liu et al. [80]	Multicenter cohort study	2020	Four sets of microbiome samples were obtained: 131 samples from a Chinese ICU cohort; 264 samples from a healthy Chinese cohort; 129 samples from an American ICU cohort; and 26 samples from a healthy American cohort.	While Enterococcus made up the majority of ICU-enterotype II (ICU E2), Bacteroides and an unknown strain of Enterobacteriaceae made up the majority of ICU-enterotype I (ICU E1). For ICU E1, septic shock was more likely to happen with APACHE II values greater than 18.
Shoji et al. [81]	Multicenter, prospective, observational study	2022	400 patients will be enrolled prospectively.	This study uses artificial intelligence to identify the precise makeup of the gut microbiome or combination of gut microbiome containing a real predictive biomarker of therapeutic response to immunotherapy in lung cancer patients. It is scheduled to conclude in September 2024, 12 months after the last person is recruited.

LOS = late-onset sepsis, CoNS = Coagulase-negative staphylococci, ICU = Intensive Care Unit.

**Table 4:** Works on therapeutic opportunities.

Authors	Type	Year	Subjects	Findings
Plantinga et al. [96]	Randomized controlled trial	2020	In six European nations, thirteen intensive care units, and 8665 individuals.	In mechanically ventilated ICU patients, SDD correlated with more remission and less acquisition of 3GCR-E and CR-GNB in the rectum than SC. The adjusted cause-specific hazard ratios (CSHR) for eradication of rectal carriage for SDD were 1.76 (95% CI 1.31–2.36) for 3GCR-E and 3.17 (95% CI 1.60–6.29) for CR-GNB compared with SC.
Rao et al. [98]	Meta-analysis	2016	Results of 37 RCTs (N = 9416).	Showed probiotics significantly decreased the risk of LOS (675/4852 [13.9%] vs. 744/4564 [16.3%]; *p* = 0.007).
Besselink et al. [100]	Randomized controlled trial	2009	Urine samples were obtained from 141 patients 24 to 48 h following the initiation of probiotic or placebo medication, and 7 days later.	This combination of probiotic strains as a prophylactic treatment decreased bacterial translocation, but was related to higher bacterial translocation and enterocyte damage among individuals with organ failure. Probiotic prophylaxis was associated with an increase in I-FABP (median 362 vs. 199 pg/mL; *p* = 0.02), most evidently in patients with organ failure (*p* = 0.001).
Johnstone et al. [101]	Randomized controlled trial	2021	In 44 ICUs in Canada, the United States, and Saudi Arabia enrolling 2653 adults predicted to require mechanical ventilation for at least 72 h.	In critically ill patients on mechanical ventilation, the use of the probiotic Lactobacillus rhamnosus GG did not show a relevant impact on the incidence of ventilator-associated pneumonia when compared to a placebo. VAP developed among 289 of 1318 patients (21.9%) receiving probiotics vs. 284 of 1332 controls (21.3%); (95% CI, 0.87–1.22; *p* = 0.73).
Wei et al. [111]	Case Reports	2016	Upon admission, a 65-year-old man was diagnosed with cerebellar hemorrhage, while an 84-year-old man was diagnosed with cerebral infarction. Both patients subsequently developed multiple organ dysfunction syndrome (MODS), septic shock, and severe watery diarrhea.	The results from treating both with fecal microbiota transplantation (FMT) suggest that reestablishing the intestinal microbiota barrier can help resolve the infection.
Ianiro et al. [116]	Randomized clinical trial	2018	A total of 56 participants were enrolled, with 28 assigned to each treatment group.	Twenty-one patients in the FMT-S group and 28 patients in the FMT-M group were cured (75% vs. 100%, respectively, *p* = 0.01).
DeFilipp et al. [117]	Case reports	2019	Two patients linked to the same stool donor by means of genomic sequencing.	Following FMT in two separate clinical trials, bacteremia caused by extended-spectrum beta-lactamase (ESBL)-producing *Escherichia coli* developed. One of the patients did not survive.

3GCR-E = third-generation cephalosporin-resistant Enterobacterales, CR-GNB = carbapenem-resistant gram-negative bacteria, SC = standard care, LOS = late-onset sepsis, FMT = fecal microbiota transplantation, FMT-S = single fecal infusion, FMT-M = multiple fecal infusion.

## Data Availability

The articles cited in this paper are available on PubMed^®^, UpToDate^®^ and Cochrane^®^.

## References

[1] Ianiro G., Iorio A., Porcari S., Masucci L., Sanguinetti M., Perno C.F., Gasbarrini A., Putignani L., Cammarota G. (2022). How the gut parasitome affects human health. Ther. Adv. Gastroenterol..

[2] De Siena M., Laterza L., Matteo M.V., Mignini I., Schepis T., Rizzatti G., Ianiro G., Rinninella E., Cintoni M., Gasbarrini A. (2021). Gut and Reproductive Tract Microbiota Adaptation during Pregnancy: New Insights for Pregnancy-Related Complications and Therapy. Microorganisms.

[3] Rinninella E., Raoul P., Cintoni M., Franceschi F., Miggiano G.A.D., Gasbarrini A., Mele M.C. (2019). What is the Healthy Gut Microbiota Composition? A Changing Ecosystem across Age, Environment, Diet, and Diseases. Microorganisms.

[4] Hiippala K., Jouhten H., Ronkainen A., Hartikainen A., Kainulainen V., Jalanka J., Satokari R. (2018). The Potential of Gut Commensals in Reinforcing Intestinal Barrier Function and Alleviating Inflammation. Nutrients.

[5] Portincasa P., Bonfrate L., Vacca M., De Angelis M., Farella I., Lanza E., Khalil M., Wang D.Q.-H., Sperandio M., Di Ciaula A. (2022). Gut Microbiota and Short Chain Fatty Acids: Implications in Glucose Homeostasis. Int. J. Mol. Sci..

[6] Jian Z., Zeng L., Xu T., Sun S., Yan S., Zhao S., Su Z., Ge C., Zhang Y., Jia J. (2022). The intestinal microbiome associated with lipid metabolism and obesity in humans and animals. J. Appl. Microbiol..

[7] Bibbò S., Ianiro G., Giorgio V., Scaldaferri F., Masucci L., Gasbarrini A., Cammarota G. (2016). The role of diet on gut microbiota composition. Eur. Rev. Med. Pharmacol. Sci..

[8] Porcari S., Fusco W., Spivak I., Fiorani M., Gasbarrini A., Elinav E., Cammarota G., Ianiro G. (2024). Fine-tuning the gut ecosystem: The current landscape and outlook of artificial microbiome therapeutics. Lancet Gastroenterol. Hepatol..

[9] Singer M., Deutschman C.S., Seymour C.W., Shankar-Hari M., Annane D., Bauer M., Bellomo R., Bernard G.R., Chiche J.-D., Coopersmith C.M. (2016). The Third International Consensus Definitions for Sepsis and Septic Shock (Sepsis-3). JAMA.

[10] Purcarea A., Sovaila S. (2020). Sepsis, a 2020 review for the internist. Rom. J. Intern. Med..

[11] Cecconi M., Evans L., Levy M., Rhodes A. (2018). Sepsis and septic shock. Lancet.

[12] Fleischmann-Struzek C., Mellhammar L., Rose N., Cassini A., Rudd K.E., Schlattmann P., Allegranzi B., Reinhart K. (2020). Incidence and mortality of hospital- and ICU-treated sepsis: Results from an updated and expanded systematic review and meta-analysis. Intensive Care Med..

[13] Rudd K.E., Johnson S.C., Agesa K.M., Shackelford K.A., Tsoi D., Kievlan D.R., Colombara D.V., Ikuta K.S., Kissoon N., Finfer S. (2020). Global, regional, and national sepsis incidence and mortality, 1990–2017: Analysis for the Global Burden of Disease Study. Lancet.

[14] Mohamed Elfadil O., Mundi M.S., Abdelmagid M.G., Patel A., Patel N., Martindale R. (2023). Butyrate: More Than a Short Chain Fatty Acid. Curr. Nutr. Rep..

[15] Haak B.W., Argelaguet R., Kinsella C.M., Kullberg R.F.J., Lankelma J.M., Deijs M., Klein M., Jebbink M.F., Hugenholtz F., Kostidis S. (2021). Integrative Transkingdom Analysis of the Gut Microbiome in Antibiotic Perturbation and Critical Illness. MSystems.

[16] Zaborin A., Smith D., Garfield K., Quensen J., Shakhsheer B., Kade M., Tirrell M., Tiedje J., Gilbert J.A., Zaborina O. (2014). Membership and Behavior of Ultra-Low-Diversity Pathogen Communities Present in the Gut of Humans during Prolonged Critical Illness. MBio.

[17] Belkaid Y., Harrison O.J. (2017). Homeostatic Immunity and the Microbiota. Immunity.

[18] Arpaia N., Campbell C., Fan X., Dikiy S., Van Der Veeken J., deRoos P., Liu H., Cross J.R., Pfeffer K., Coffer P.J. (2013). Metabolites produced by commensal bacteria promote peripheral regulatory T-cell generation. Nature.

[19] Schulthess J., Pandey S., Capitani M., Rue-Albrecht K.C., Arnold I., Franchini F., Chomka A., Ilott N.E., Johnston D.G.W., Pires E. (2019). The Short Chain Fatty Acid Butyrate Imprints an Antimicrobial Program in Macrophages. Immunity.

[20] Piccioni A., Franza L., Brigida M., Zanza C., Torelli E., Petrucci M., Nicolò R., Covino M., Candelli M., Saviano A. (2021). Gut Microbiota and Acute Diverticulitis: Role of Probiotics in Management of This Delicate Pathophysiological Balance. J. Pers. Med..

[21] Wang F., Liu J., Weng T., Shen K., Chen Z., Yu Y., Huang Q., Wang G., Liu Z., Jin S. (2017). The Inflammation Induced by Lipopolysaccharide can be Mitigated by Short-chain Fatty Acid, Butyrate, through Upregulation of IL-10 in Septic Shock. Scand. J. Immunol..

[22] Yamada T., Shimizu K., Ogura H., Asahara T., Nomoto K., Yamakawa K., Hamasaki T., Nakahori Y., Ohnishi M., Kuwagata Y. (2015). Rapid and Sustained Long-Term Decrease of Fecal Short-Chain Fatty Acids in Critically Ill Patients With Systemic Inflammatory Response Syndrome. JPEN J. Parenter. Enteral Nutr..

[23] Martin-Gallausiaux C., Marinelli L., Blottière H.M., Larraufie P., Lapaque N. (2021). SCFA: Mechanisms and functional importance in the gut. Proc. Nutr. Soc..

[24] McDonald B., Zucoloto A.Z., Yu I.-L., Burkhard R., Brown K., Geuking M.B., McCoy K.D. (2020). Programing of an Intravascular Immune Firewall by the Gut Microbiota Protects against Pathogen Dissemination during Infection. Cell Host Microbe.

[25] Van Der Hee B., Wells J.M. (2021). Microbial Regulation of Host Physiology by Short-chain Fatty Acids. Trends Microbiol..

[26] Wang Y., Deng H., Xiao L., Pan Y. (2024). Escherichia coli Nissle 1917 Protects against Sepsis-Induced Intestinal Damage by Regulating the SCFA/GPRs Signaling Pathway. Microorganisms.

[27] David L.A., Maurice C.F., Carmody R.N., Gootenberg D.B., Button J.E., Wolfe B.E., Ling A.V., Devlin A.S., Varma Y., Fischbach M.A. (2014). Diet rapidly and reproducibly alters the human gut microbiome. Nature.

[28] Hill A., Elke G., Weimann A. (2021). Nutrition in the Intensive Care Unit—A Narrative Review. Nutrients.

[29] Grillo-Ardila C.F., Tibavizco-Palacios D., Triana L.C., Rugeles S.J., Vallejo-Ortega M.T., Calderón-Franco C.H., Ramírez-Mosquera J.J. (2024). Early Enteral Nutrition (within 48 h) for Patients with Sepsis or Septic Shock: A Systematic Review and Meta-Analysis. Nutrients.

[30] Moon S.J., Ko R.-E., Park C.-M., Suh G.Y., Hwang J., Chung C.R. (2023). The Effectiveness of Early Enteral Nutrition on Clinical Outcomes in Critically Ill Sepsis Patients: A Systematic Review. Nutrients.

[31] Kaewdech A., Sripongpun P., Wetwittayakhlang P., Churuangsuk C. (2022). The effect of fiber supplementation on the prevention of diarrhea in hospitalized patients receiving enteral nutrition: A meta-analysis of randomized controlled trials with the GRADE assessment. Front. Nutr..

[32] Huwiler V.V., Scalise M., Schönenberger K.A., Mühlebach S., Stanga Z., Balmer M.L. (2023). The Role of Dietary Fibre in Enteral Nutrition in Sepsis Prevention and Therapy: A Narrative Review. Nutrients.

[33] Lopez-Delgado J.C., Grau-Carmona T., Trujillano-Cabello J., García-Fuentes C., Mor-Marco E., Bordeje-Laguna M.L., Portugal-Rodriguez E., Lorencio-Cardenas C., Vera-Artazcoz P., Macaya-Redin L. (2022). The Effect of Enteral Immunonutrition in the Intensive Care Unit: Does It Impact on Outcomes?. Nutrients.

[34] Schreiber F., Balas I., Robinson M.J., Bakdash G. (2024). Border Control: The Role of the Microbiome in Regulating Epithelial Barrier Function. Cells.

[35] McArthur S. (2023). Regulation of Physiological Barrier Function by the Commensal Microbiota. Life.

[36] Saleri R., Borghetti P., Ravanetti F., Cavalli V., Ferrari L., De Angelis E., Andrani M., Martelli P. (2022). Effects of different short-chain fatty acids (SCFA) on gene expression of proteins involved in barrier function in IPEC-J2. Porc. Health Manag..

[37] Ghosh S., Whitley C.S., Haribabu B., Jala V.R. (2021). Regulation of Intestinal Barrier Function by Microbial Metabolites. Cell. Mol. Gastroenterol. Hepatol..

[38] Rao J.N., Xiao L., Wang J.-Y. (2020). Polyamines in Gut Epithelial Renewal and Barrier Function. Physiology.

[39] Chen Y., Chen H., Ding J., Stanton C., Ross R.P., Zhao J., Zhang H., Yang B., Chen W. (2021). Bifidobacterium longum Ameliorates Dextran Sulfate Sodium-Induced Colitis by Producing Conjugated Linoleic Acid, Protecting Intestinal Mechanical Barrier, Restoring Unbalanced Gut Microbiota, and Regulating the Toll-Like Receptor-4/Nuclear Factor-κB Signaling Pathway. J. Agric. Food Chem..

[40] Ren Q., Yang B., Zhang H., Ross R.P., Stanton C., Chen H., Chen W. (2020). c9, t11, c15-CLNA and t9, t11, c15-CLNA from Lactobacillus plantarum ZS2058 Ameliorate Dextran Sodium Sulfate-Induced Colitis in Mice. J. Agric. Food Chem..

[41] Zhan L., Zheng J., Meng J., Fu D., Pang L., Ji C. (2022). Toll-like receptor 4 deficiency alleviates lipopolysaccharide-induced intestinal barrier dysfunction. Biomed. Pharmacother..

[42] Gu M.J., Song S.K., Lee I.K., Ko S., Han S.E., Bae S., Ji S.Y., Park B.-C., Song K.-D., Lee H.-K. (2016). Barrier protection via Toll-like receptor 2 signaling in porcine intestinal epithelial cells damaged by deoxynivalnol. Vet. Res..

[43] Yoseph B.P., Klingensmith N.J., Liang Z., Breed E.R., Burd E.M., Mittal R., Dominguez J.A., Petrie B., Ford M.L., Coopersmith C.M. (2016). Mechanisms of Intestinal Barrier Dysfunction in Sepsis. Shock.

[44] Jung C., Meinzer U., Montcuquet N., Thachil E., Château D., Thiébaut R., Roy M., Alnabhani Z., Berrebi D., Dussaillant M. (2012). Yersinia pseudotuberculosis disrupts intestinal barrier integrity through hematopoietic TLR-2 signaling. J. Clin. Investig..

[45] Lorentz C.A., Liang Z., Meng M., Chen C.-W., Yoseph B.P., Breed E.R., Mittal R., Klingensmith N.J., Farris A.B., Burd E.M. (2017). Myosin light chain kinase knockout improves gut barrier function and confers a survival advantage in polymicrobial sepsis. Mol. Med. Camb. Mass.

[46] Schirmer M., Smeekens S.P., Vlamakis H., Jaeger M., Oosting M., Franzosa E.A., Ter Horst R., Jansen T., Jacobs L., Bonder M.J. (2016). Linking the Human Gut Microbiome to Inflammatory Cytokine Production Capacity. Cell.

[47] Khosravi A., Yáñez A., Price J.G., Chow A., Merad M., Goodridge H.S., Mazmanian S.K. (2014). Gut microbiota promote hematopoiesis to control bacterial infection. Cell Host Microbe.

[48] Zhang D., Chen G., Manwani D., Mortha A., Xu C., Faith J.J., Burk R.D., Kunisaki Y., Jang J.-E., Scheiermann C. (2015). Neutrophil ageing is regulated by the microbiome. Nature.

[49] Wilmore J.R., Gaudette B.T., Gomez Atria D., Hashemi T., Jones D.D., Gardner C.A., Cole S.D., Misic A.M., Beiting D.P., Allman D. (2018). Commensal Microbes Induce Serum IgA Responses that Protect against Polymicrobial Sepsis. Cell Host Microbe.

[50] Zeng M.Y., Cisalpino D., Varadarajan S., Hellman J., Warren H.S., Cascalho M., Inohara N., Núñez G. (2016). Gut Microbiota-Induced Immunoglobulin G Controls Systemic Infection by Symbiotic Bacteria and Pathogens. Immunity.

[51] Heilbronner S., Krismer B., Brötz-Oesterhelt H., Peschel A. (2021). The microbiome-shaping roles of bacteriocins. Nat. Rev. Microbiol..

[52] Behrens H.M., Six A., Walker D., Kleanthous C. (2017). The therapeutic potential of bacteriocins as protein antibiotics. Emerg. Top. Life Sci..

[53] Schuijt T.J., Lankelma J.M., Scicluna B.P., De Sousa E Melo F., Roelofs J.J.T.H., De Boer J.D., Hoogendijk A.J., De Beer R., De Vos A., Belzer C. (2016). The gut microbiota plays a protective role in the host defence against pneumococcal pneumonia. Gut.

[54] Lou X., Xue J., Shao R., Yang Y., Ning D., Mo C., Wang F., Chen G. (2023). Fecal microbiota transplantation and short-chain fatty acids reduce sepsis mortality by remodeling antibiotic-induced gut microbiota disturbances. Front. Immunol..

[55] Livanos A.E., Snider E.J., Whittier S., Chong D.H., Wang T.C., Abrams J.A., Freedberg D.E. (2018). Rapid gastrointestinal loss of Clostridial Clusters IV and XIVa in the ICU associates with an expansion of gut pathogens. PLoS ONE.

[56] Wolff N.S., Hugenholtz F., Wiersinga W.J. (2018). The emerging role of the microbiota in the ICU. Crit. Care Lond. Engl..

[57] Lankelma J.M., van Vught L.A., Belzer C., Schultz M.J., van der Poll T., de Vos W.M., Wiersinga W.J. (2017). Critically ill patients demonstrate large interpersonal variation in intestinal microbiota dysregulation: A pilot study. Intensive Care Med..

[58] Ubeda C., Taur Y., Jenq R.R., Equinda M.J., Son T., Samstein M., Viale A., Socci N.D., Van Den Brink M.R.M., Kamboj M. (2010). Vancomycin-resistant Enterococcus domination of intestinal microbiota is enabled by antibiotic treatment in mice and precedes bloodstream invasion in humans. J. Clin. Investig..

[59] Patrier J., Villageois-Tran K., Szychowiak P., Ruckly S., Gschwind R., Wicky P.-H., Gueye S., Armand-Lefevre L., Marzouk M., Sonneville R. (2022). Oropharyngeal and intestinal concentrations of opportunistic pathogens are independently associated with death of SARS-CoV-2 critically ill adults. Crit. Care.

[60] Hayakawa M., Asahara T., Henzan N., Murakami H., Yamamoto H., Mukai N., Minami Y., Sugano M., Kubota N., Uegaki S. (2011). Dramatic Changes of the Gut Flora Immediately After Severe and Sudden Insults. Dig. Dis. Sci..

[61] Zanza C., Romenskaya T., Thangathurai D., Ojetti V., Saviano A., Abenavoli L., Robba C., Cammarota G., Franceschi F., Piccioni A. (2022). Microbiome in Critical Care: An Unconventional and Unknown Ally. Curr. Med. Chem..

[62] Perrone E.E., Jung E., Breed E., Dominguez J.A., Liang Z., Clark A.T., Dunne W.M., Burd E.M., Coopersmith C.M. (2012). Mechanisms of methicillin-resistant Staphylococcus aureus pneumonia-induced intestinal epithelial apoptosis. Shock.

[63] Taur Y., Xavier J.B., Lipuma L., Ubeda C., Goldberg J., Gobourne A., Lee Y.J., Dubin K.A., Socci N.D., Viale A. (2012). Intestinal Domination and the Risk of Bacteremia in Patients Undergoing Allogeneic Hematopoietic Stem Cell Transplantation. Clin. Infect. Dis..

[64] Niu X., Daniel S., Kumar D., Ding E.Y., Savani R.C., Koh A.Y., Mirpuri J. (2020). Transient neonatal antibiotic exposure increases susceptibility to late-onset sepsis driven by microbiota-dependent suppression of type 3 innate lymphoid cells. Sci. Rep..

[65] Prescott H.C., Dickson R.P., Rogers M.A.M., Langa K.M., Iwashyna T.J. (2015). Hospitalization Type and Subsequent Severe Sepsis. Am. J. Respir. Crit. Care Med..

[66] Dickson R.P., Singer B.H., Newstead M.W., Falkowski N.R., Erb-Downward J.R., Standiford T.J., Huffnagle G.B. (2016). Enrichment of the lung microbiome with gut bacteria in sepsis and the acute respiratory distress syndrome. Nat. Microbiol..

[67] Singer J.R., Blosser E.G., Zindl C.L., Silberger D.J., Conlan S., Laufer V.A., DiToro D., Deming C., Kumar R., Morrow C.D. (2019). Preventing dysbiosis of the neonatal mouse intestinal microbiome protects against late-onset sepsis. Nat. Med..

[68] De Lastours V., Goulenok T., Guérin F., Jacquier H., Eyma C., Chau F., Cattoir V., Fantin B. (2018). Ceftriaxone promotes the emergence of AmpC-overproducing Enterobacteriaceae in gut microbiota from hospitalized patients. Eur. J. Clin. Microbiol. Infect. Dis..

[69] Smits W.K., Lyras D., Lacy D.B., Wilcox M.H., Kuijper E.J. (2016). Clostridium difficile infection. Nat. Rev. Dis. Primer.

[70] Zimmermann P., Curtis N. (2019). The effect of antibiotics on the composition of the intestinal microbiota—A systematic review. J. Infect..

[71] Freedberg D.E., Zhou M.J., Cohen M.E., Annavajhala M.K., Khan S., Moscoso D.I., Brooks C., Whittier S., Chong D.H., Uhlemann A.-C. (2018). Pathogen colonization of the gastrointestinal microbiome at intensive care unit admission and risk for subsequent death or infection. Intensive Care Med..

[72] Zhao X., Jiang Z., Yang F., Wang Y., Gao X., Wang Y., Chai X., Pan G., Zhu Y. (2016). Sensitive and Simplified Detection of Antibiotic Influence on the Dynamic and Versatile Changes of Fecal Short-Chain Fatty Acids. PLoS ONE.

[73] Suez J., Zmora N., Zilberman-Schapira G., Mor U., Dori-Bachash M., Bashiardes S., Zur M., Regev-Lehavi D., Ben-Zeev Brik R., Federici S. (2018). Post-Antibiotic Gut Mucosal Microbiome Reconstitution Is Impaired by Probiotics and Improved by Autologous FMT. Cell.

[74] Stein-Thoeringer C.K., Nichols K.B., Lazrak A., Docampo M.D., Slingerland A.E., Slingerland J.B., Clurman A.G., Armijo G., Gomes A.L.C., Shono Y. (2019). Lactose drives *Enterococcus* expansion to promote graft-versus-host disease. Science.

[75] Shimizu K., Ogura H., Asahara T., Nomoto K., Morotomi M., Nakahori Y., Osuka A., Yamano S., Goto M., Matsushima A. (2011). Gastrointestinal dysmotility is associated with altered gut flora and septic mortality in patients with severe systemic inflammatory response syndrome: A preliminary study: Dysmotility and altered gut flora in SIRS. Neurogastroenterol. Motil..

[76] El Manouni El Hassani S., Niemarkt H.J., Berkhout D.J.C., Peeters C.F.W., Hulzebos C.V., Van Kaam A.H., Kramer B.W., Van Lingen R.A., Jenken F., De Boode W.P. (2021). Profound Pathogen-Specific Alterations in Intestinal Microbiota Composition Precede Late-Onset Sepsis in Preterm Infants: A Longitudinal, Multicenter, Case-Control Study. Clin. Infect. Dis..

[77] Marascio N., Scarlata G.G.M., Romeo F., Cicino C., Trecarichi E.M., Quirino A., Torti C., Matera G., Russo A. (2023). The Role of Gut Microbiota in the Clinical Outcome of Septic Patients: State of the Art and Future Perspectives. Int. J. Mol. Sci..

[78] Graspeuntner S., Waschina S., Künzel S., Twisselmann N., Rausch T.K., Cloppenborg-Schmidt K., Zimmermann J., Viemann D., Herting E., Göpel W. (2019). Gut Dysbiosis With Bacilli Dominance and Accumulation of Fermentation Products Precedes Late-onset Sepsis in Preterm Infants. Clin. Infect. Dis..

[79] Stoma I., Littmann E.R., Peled J.U., Giralt S., Van Den Brink M.R.M., Pamer E.G., Taur Y. (2021). Compositional Flux Within the Intestinal Microbiota and Risk for Bloodstream Infection With Gram-negative Bacteria. Clin. Infect. Dis..

[80] Liu W., Cheng M., Li J., Zhang P., Fan H., Hu Q., Han M., Su L., He H., Tong Y. (2020). Classification of the Gut Microbiota of Patients in Intensive Care Units During Development of Sepsis and Septic Shock. Genom. Proteom. Bioinform..

[81] Shoji F., Yamashita T., Kinoshita F., Takamori S., Fujishita T., Toyozawa R., Ito K., Yamazaki K., Nakashima N., Okamoto T. (2022). Artificial intelligence-derived gut microbiome as a predictive biomarker for therapeutic response to immunotherapy in lung cancer: Protocol for a multicentre, prospective, observational study. BMJ Open.

[82] Alam A., Neish A. (2018). Role of gut microbiota in intestinal wound healing and barrier function. Tissue Barriers.

[83] Chakaroun R.M., Massier L., Kovacs P. (2020). Gut Microbiome, Intestinal Permeability, and Tissue Bacteria in Metabolic Disease: Perpetrators or Bystanders?. Nutrients.

[84] Ohland C.L., Macnaughton W.K. (2010). Probiotic bacteria and intestinal epithelial barrier function. Am. J. Physiol. Gastrointest. Liver Physiol..

[85] Bischoff S.C., Barbara G., Buurman W., Ockhuizen T., Schulzke J.-D., Serino M., Tilg H., Watson A., Wells J.M. (2014). Intestinal permeability--a new target for disease prevention and therapy. BMC Gastroenterol..

[86] Schroeder B.O., Bäckhed F. (2016). Signals from the gut microbiota to distant organs in physiology and disease. Nat. Med..

[87] Longhitano Y., Zanza C., Thangathurai D., Taurone S., Kozel D., Racca F., Audo A., Ravera E., Migneco A., Piccioni A. (2021). Gut Alterations in Septic Patients: A Biochemical Literature Review. Rev. Recent Clin. Trials.

[88] Luiking Y.C., Poeze M., Ramsay G., Deutz N.E. (2009). Reduced citrulline production in sepsis is related to diminished de novo arginine and nitric oxide production. Am. J. Clin. Nutr..

[89] Fragkos K.C., Forbes A. (2018). Citrulline as a marker of intestinal function and absorption in clinical settings: A systematic review and meta-analysis. United Eur. Gastroenterol. J..

[90] Kong C., Li S.-M., Yang H., Xiao W.-D., Cen Y.-Y., Wu Y., Li W.-M., Sun D.-L., Xu P.-Y. (2019). Screening and combining serum biomarkers to improve their diagnostic performance in the detection of intestinal barrier dysfunction in patients after major abdominal surgery. Ann. Transl. Med..

[91] Cai Y., Gong D., Xiang T., Zhang X., Pan J. (2024). Markers of intestinal barrier damage in patients with chronic insomnia disorder. Front. Psychiatry.

[92] Efremova I., Maslennikov R., Medvedev O., Kudryavtseva A., Avdeeva A., Krasnov G., Romanikhin F., Diatroptov M., Fedorova M., Poluektova E. (2024). Gut Microbiota and Biomarkers of Intestinal Barrier Damage in Cirrhosis. Microorganisms.

[93] Guerville M., Leroy A., Sinquin A., Laugerette F., Michalski M.-C., Boudry G. (2017). Western-diet consumption induces alteration of barrier function mechanisms in the ileum that correlates with metabolic endotoxemia in rats. Am. J. Physiol. Endocrinol. Metab..

[94] Rezaie A., Buresi M., Lembo A., Lin H., McCallum R., Rao S., Schmulson M., Valdovinos M., Zakko S., Pimentel M. (2017). Hydrogen and Methane-Based Breath Testing in Gastrointestinal Disorders: The North American Consensus. Am. J. Gastroenterol..

[95] Wittekamp B.H.J., Oostdijk E.A.N., Cuthbertson B.H., Brun-Buisson C., Bonten M.J.M. (2020). Selective decontamination of the digestive tract (SDD) in critically ill patients: A narrative review. Intensive Care Med..

[96] Plantinga N.L., Wittekamp B.H.J., Brun-Buisson C., Bonten M.J.M., Cooper B.S., Coll P., Lopez-Contreras J., Mancebo J., Wise M.P., Morgan M.P.G. (2020). The effects of topical antibiotics on eradication and acquisition of third-generation cephalosporin and carbapenem-resistant Gram-negative bacteria in ICU patients; a post hoc analysis from a multicentre cluster-randomized trial. Clin. Microbiol. Infect..

[97] Hill C., Guarner F., Reid G., Gibson G.R., Merenstein D.J., Pot B., Morelli L., Canani R.B., Flint H.J., Salminen S. (2014). The International Scientific Association for Probiotics and Prebiotics consensus statement on the scope and appropriate use of the term probiotic. Nat. Rev. Gastroenterol. Hepatol..

[98] Rao S.C., Athalye-Jape G.K., Deshpande G.C., Simmer K.N., Patole S.K. (2016). Probiotic Supplementation and Late-Onset Sepsis in Preterm Infants: A Meta-analysis. Pediatrics.

[99] Bassetti M., Bandera A., Gori A. (2020). Therapeutic Potential of the Gut Microbiota in the Management of Sepsis. Crit. Care.

[100] Besselink M.G., Van Santvoort H.C., Renooij W., De Smet M.B., Boermeester M.A., Fischer K., Timmerman H.M., Ahmed Ali U., Cirkel G.A., Bollen T.L. (2009). Intestinal Barrier Dysfunction in a Randomized Trial of a Specific Probiotic Composition in Acute Pancreatitis. Ann. Surg..

[101] Johnstone J., Meade M., Lauzier F., Marshall J., Duan E., Dionne J., Arabi Y.M., Heels-Ansdell D., Thabane L., Lamarche D. (2021). Effect of Probiotics on Incident Ventilator-Associated Pneumonia in Critically Ill Patients: A Randomized Clinical Trial. JAMA.

[102] Gibson G.R., Hutkins R., Sanders M.E., Prescott S.L., Reimer R.A., Salminen S.J., Scott K., Stanton C., Swanson K.S., Cani P.D. (2017). Expert consensus document: The International Scientific Association for Probiotics and Prebiotics (ISAPP) consensus statement on the definition and scope of prebiotics. Nat. Rev. Gastroenterol. Hepatol..

[103] Davani-Davari D., Negahdaripour M., Karimzadeh I., Seifan M., Mohkam M., Masoumi S., Berenjian A., Ghasemi Y. (2019). Prebiotics: Definition, Types, Sources, Mechanisms, and Clinical Applications. Foods.

[104] Krga I. (2022). Therapeutics and microbiota. Microbiota Health Dis..

[105] Swanson K.S., Gibson G.R., Hutkins R., Reimer R.A., Reid G., Verbeke K., Scott K.P., Holscher H.D., Azad M.B., Delzenne N.M. (2020). The International Scientific Association for Probiotics and Prebiotics (ISAPP) consensus statement on the definition and scope of synbiotics. Nat. Rev. Gastroenterol. Hepatol..

[106] Wang K., Zeng Q., Li K., Wang Y., Wang L., Sun M., Zeng J., Jiang H. (2022). Efficacy of probiotics or synbiotics for critically ill adult patients: A systematic review and meta-analysis of randomized controlled trials. Burns Trauma.

[107] Salminen S., Collado M.C., Endo A., Hill C., Lebeer S., Quigley E.M.M., Sanders M.E., Shamir R., Swann J.R., Szajewska H. (2021). The International Scientific Association of Probiotics and Prebiotics (ISAPP) consensus statement on the definition and scope of postbiotics. Nat. Rev. Gastroenterol. Hepatol..

[108] Nataraj B.H., Ali S.A., Behare P.V., Yadav H. (2020). Postbiotics-parabiotics: The new horizons in microbial biotherapy and functional foods. Microb. Cell Factories.

[109] Patel R.M., Denning P.W. (2013). Therapeutic use of prebiotics, probiotics, and postbiotics to prevent necrotizing enterocolitis: What is the current evidence?. Clin. Perinatol..

[110] Lou X., Xue J., Shao R., Mo C., Wang F., Chen G. (2023). Postbiotics as potential new therapeutic agents for sepsis. Burns Trauma.

[111] Wei Y., Yang J., Wang J., Yang Y., Huang J., Gong H., Cui H., Chen D. (2016). Successful treatment with fecal microbiota transplantation in patients with multiple organ dysfunction syndrome and diarrhea following severe sepsis. Crit. Care.

[112] Piccioni A., Rosa F., Manca F., Pignataro G., Zanza C., Savioli G., Covino M., Ojetti V., Gasbarrini A., Franceschi F. (2022). Gut Microbiota and Clostridium difficile: What We Know and the New Frontiers. Int. J. Mol. Sci..

[113] Jang H.R., Gandolfo M.T., Ko G.J., Satpute S., Racusen L., Rabb H. (2009). Early exposure to germs modifies kidney damage and inflammation after experimental ischemia-reperfusion injury. Am. J. Physiol.-Ren. Physiol..

[114] Andrade-Oliveira V., Amano M.T., Correa-Costa M., Castoldi A., Felizardo R.J.F., De Almeida D.C., Bassi E.J., Moraes-Vieira P.M., Hiyane M.I., Rodas A.C.D. (2015). Gut Bacteria Products Prevent AKI Induced by Ischemia-Reperfusion. J. Am. Soc. Nephrol..

[115] Rabb H., Pluznick J., Noel S. (2018). The Microbiome and Acute Kidney Injury. Nephron.

[116] Ianiro G., Masucci L., Quaranta G., Simonelli C., Lopetuso L.R., Sanguinetti M., Gasbarrini A., Cammarota G. (2018). Randomised clinical trial: Faecal microbiota transplantation by colonoscopy plus vancomycin for the treatment of severe refractory *Clostridium difficile* infection—Single versus multiple infusions. Aliment. Pharmacol. Ther..

[117] DeFilipp Z., Bloom P.P., Torres Soto M., Mansour M.K., Sater M.R.A., Huntley M.H., Turbett S., Chung R.T., Chen Y.-B., Hohmann E.L. (2019). Drug-Resistant *E. coli* Bacteremia Transmitted by Fecal Microbiota Transplant. N. Engl. J. Med..

[118] Mullish B.H., Alexander J.L., Segal J.P. (2021). Microbiota and faecal microbiota transplant. Microbiota Health Dis..

